# Long-Lasting Efficacy of Radio Electric Asymmetric Conveyer Neuromodulation Treatment on Functional Dysmetria, an Adaptive Motor Behavior

**DOI:** 10.7759/cureus.25768

**Published:** 2022-06-08

**Authors:** Vania Fontani, Arianna Rinaldi, Chiara Rinaldi, Laura Araldi, Alida Azzarà, Antonio M Carta, Nicoletta Casale, Alessandro Castagna, Maurizio Del Medico, Maurizio Di Stasio, Marina Facchini, Monica Greco, Savino LaMarca, Giovanni Loro, Anna Marrone, Alessandra Palattella, Giulio Pellegata, Daniele Ruini, Corrado Schmitt, Franco Vianini, Margherita Maioli, Carlo Ventura, Franco Caltabiano, Adriano J Bueno, Amélia Fugino Matuoka, Edison Massahiro Nabechima, Fabio A Bechelli, Fabricio da Silveira Bossi, Greice C Nitschke Fontana, Jaques Finkielsztejn, José Alfredo Coelho Pereira, Juarez Nunes Callegaro, Kleiner Vasconcelos Pinheiro, Lara R Ferreira Alves, Marcelo Kodja Daguer, Márcia C Marins Martins, Mauricio Bezerra Uliana, Nelson Knop Zisman, Paulo Cezar Schütz, Paulo R Fochesato, Pollyanna Celso Felipe de Castro, Rosa M Tanaka Nabechima, Roseli B Randon, Salvatore Rinaldi

**Affiliations:** 1 Research, Rinaldi Fontani Foundation, Florence, ITA; 2 Regenerative Medicine, Rinaldi Fontani Institute, Florence, ITA; 3 Biomedical Sciences, University of Sassari, Sassari, ITA; 4 Neuroscience, Psychology, Drug Area and Child Health (NEUROFARBA), University of Florence, Florence, ITA; 5 Internal Medicine, International Scientific Society of Neuro Psycho Physical Optimization with REAC Technology, Florence, ITA; 6 Obstetrics and Gynecology, International Scientific Society of Neuro Psycho Physical Optimization with REAC Technology, Florence, ITA; 7 Medical Physics, International Scientific Society of Neuro Psycho Physical Optimization with REAC Technology, Florence, ITA; 8 Neurology, International Scientific Society of Neuro Psycho Physical Optimization with REAC Technology, Florence, ITA; 9 Geriatrics, International Scientific Society of Neuro Psycho Physical Optimization with REAC Technology, Florence, ITA; 10 Oral Medicine, International Scientific Society of Neuro Psycho Physical Optimization with REAC Technology, Florence, ITA; 11 Diabetes and Endocrinology, International Scientific Society of Neuro Psycho Physical Optimization with REAC Technology, Florence, ITA; 12 Psychiatry, International Scientific Society of Neuro Psycho Physical Optimization with REAC Technology, Florence, ITA; 13 Anesthesiology, International Scientific Society of Neuro Psycho Physical Optimization with REAC Technology, Florence, ITA; 14 Family Medicine, International Scientific Society of Neuro Psycho Physical Optimization with REAC Technology, Florence, ITA; 15 Cardiology and Microbiology, National Laboratory of Molecular Biology and Stem Cell Engineering, National Institute of Biostructures and Biosystems, Bologna, ITA; 16 Surgery and Urology, International Scientific Society of Neuro Psycho Physical Optimization with REAC Technology, Florence, ITA; 17 Orthopaedics, International Scientific Society of Neuro Psycho Physical Optimization with REAC Technology, São Paulo, BRA; 18 Internal Medicine, International Scientific Society of Neuro Psycho Physical Optimization with REAC Technology, São Paulo, BRA; 19 Surgery, Endoscopy and Gastroenterology, International Scientific Society of Neuro Psycho Physical Optimization with REAC Technology, São Paulo, BRA; 20 Psychiatry, International Scientific Society of Neuro Psycho Physical Optimization with REAC Technology, São Paulo, BRA; 21 Integrative/Complementary Medicine, International Scientific Society of Neuro Psycho Physical Optimization with REAC Technology, São Paulo, BRA; 22 Pediatrics, International Scientific Society of Neuro Psycho Physical Optimization with REAC Technology, São Paulo, BRA; 23 Neurology, International Scientific Society of Neuro Psycho Physical Optimization with REAC Technology, São Paulo, BRA

**Keywords:** neurobiological modulation, neurostimulation, neuromodulation, functional dysmetria, radio electric asymmetric conveyor, epigenetic, neuroplasticity, neurotransmission, endogenous bioelectric activity

## Abstract

Background

Fluctuating asymmetry (FA) is widely defined as the deviation from perfect bilateral symmetry and is considered an epigenetic measure of environmental stress. Rinaldi and Fontani hypothesized that the FA morpho-functional changes originate from an adaptive motor behavior determined by functional alterations in the cerebellum and neural circuits, not caused by a lesion, but induced by environmental stress. They called this phenomenon functional dysmetria (FD). On this premise, they developed the radio electric asymmetric conveyer (REAC) technology, a neuromodulation technology aimed at optimizing the best neuro-psycho-motor strategies in relation to environmental interaction.

Aims

Previous studies showed that specific REAC neuro postural optimization (NPO) treatment can induce stable FD recovery. This study aimed to verify the duration of the NPO effect in inducing the stable FD recovery over time.

Materials and methods

Data were retrospectively collected from a population of 29,794 subjects who underwent a specific semiological FD assessment and received the NPO treatment, regardless of the pathology referred.

Results

The analysis of the data collected by the various participants in the study led us to ascertain the disappearance of FD in 100% of the cases treated, with a stability of the result detected up to 18 years after the single administration of the REAC NPO treatment.

Conclusions

The REAC NPO neurobiological modulation treatment consisting of a single administration surprisingly maintains a very long efficacy in the correction of FD. This effect can be explained as the long-lasting capacity of the NPO treatment to induce greater functional efficiency of the brain dynamics as proven in previous studies.

## Introduction

In living organisms, the loss of perfect somatic symmetry is constantly observed. This phenomenon called fluctuating asymmetry (FA) is considered an epigenetic measure of environmental stress [1]. Fluctuating asymmetry is defined as the deviation from perfect bilateral symmetry. This deviation is caused by environmental stressors, developmental instability, and genetic/epigenetic problems during development [1-4]. Therefore, both genomic and environmental or epigenetic changes can increase FA. Consequently, FA is a useful phenomenon for stress monitoring both in nature and in the laboratory.

Fluctuating asymmetry is the manifestation of a deterioration of the allostatic mechanisms of development, which can be highlighted through changes in the individual morphological symmetry [2-4]. Since FA is an omnipresent phenomenon in all living beings, its presence is not considered a pathology, but an indicator of environmental stress [1-4]. Although there is almost unanimous agreement on its meaning, the mechanisms by which FA occurs or how it can be countered have not yet been identified.

Rinaldi and Fontani have focused on the processes that result in FA. They started from the observation that to induce a morphological asymmetry such as FA, the affected structures must be subjected to a different mechanical load. In living organisms, muscles are the structures that contract and generate mechanical force that is transmitted to their insertion points [5]. If the forces generated by the muscles, particularly by the hetero-lateral ones, are asymmetrical, a difference in both pressure and traction is created. This determines the so-called piezoelectric effect [6], a phenomenon that induces variations of the endogenous bioelectric activity (EBA) [7], i.e., electro negativity/electro positivity both at the bone level and in the organic component [8,9]. A morpho-functional remodeling follows in the structures affected by the piezoelectric phenomenon [10]. In summary, the authors have hypothesized that the asymmetrical activation (dysmetria) and the consequent piezoelectric effect are the cause of those morpho-functional changes that are manifested in the FA.

The lack of motor coordination and executive precision is already defined in the literature as dysmetria and is typically identified as a manifestation of cerebellar dysfunction or lesion [11]. Dysmetria can be defined as an error in trajectory due to an abnormal range, rate, and/or force of motion as in Holmes’s [12] definition, or a disturbance of the trajectory or placement of a body part during active movement, both in range and direction, as in Textbook of Clinical Neurology (third edition) [13].

Thanks to the new knowledge on cerebellar functions, today the term dysmetria can also indicate dysmetria of thought or the inability to adapt i.e., modulate precise and adequate behaviors around an allostatic-homeostatic baseline [14]. The cerebellum plays numerous key roles in the emotional [15], cognitive, and behavioral spheres [16], and in motor control, coordination, sense of balance, learning, and some cognitive functions related to language and attention. Considering the variety and complexity of the functions it performs, it follows that the cerebellum is widely involved in adaptive phenomena [17-19].

When dysmetria can be observed in healthy subjects [20,21], it has no lesional or pathological origin. Therefore, Rinaldi and Fontani deduced that it could be the result of an adaptive dysfunction at the level of the neural circuits, such as the cerebellar ones [22], that control the “symmetry and adequacy” of movement, and the emotional, cognitive, and behavioral sphere.

Proceeding in this research, Rinaldi and Fontani developed the term and the concept of functional dysmetria (FD) [20,21,23], to describe an adaptive motor behavior [24] determined by functional alterations in the cerebellum [18,25] and neural circuits [26], induced by the experienced environmental stress or allostatic overload [19]. Similarly, the FA is the morphological manifestation of an epigenetic phenomenon and an indicator of environmental stress [1]. While in the specialized orthopedic literature, the term dysmetria is sometimes erroneously used to indicate a heteromeric measurement of the limbs or bone segments [27], Rinaldi and Fontani correlated FD to the phenomena of dysfunctional adaptive motor behavioral response to environmental stressors.

Over the years, Rinaldi and Fontani have investigated how to recognize and differentiate the presence of FD and they identified a specific motor task to assess the asymmetrical activation of symmetrical muscle groups [20,21]. This semiological procedure highlights the asymmetrical activation of both the quadriceps, during a motor task [20,21]. Once they had standardized the FD assessment, Rinaldi and Fontani focused on how to recover the dysfunctional neuro-psycho-physical adaptive mechanisms underlying FD. For this purpose, they developed the radio electric asymmetric conveyer (REAC) technology, a neurobiological modulation technology aimed at recovering the best motor and behavioral strategies in response to environmental stressors [28-35].

Since previous studies using a specific treatment of REAC technology, the neuro postural optimization (NPO), has been shown to induce the stable recovery of FD [20,21], the purpose of this study was to verify the duration of the NPO effect over time and formulate the most likely hypothesis about the verified correlation between the REAC NPO treatment and the stable duration of the recovery of the FD. This FD recovery could be explained as the REAC NPO efficacy in inducing greater functional efficiency of the brain dynamics, previously observed in functional magnetic resonance imaging (fMRI) studies [20,21,23].

## Materials and methods

Ethics

This research was conducted according to the guidelines of the Declaration of Helsinki. The Italian branch of this research was approved by the Institutional Review Board of the Centre for Developmental Biology and Reprogramming (CEDEBIOR) of the University of Sassari, Italy (approval no. 03/2021), while the Brazilian branch was approved by the Ethical Committee of Federal University of Amapá (UNIFAP) (approval no. 3.238.424 02). Informed consent was obtained from all subjects that received the NPO Treatment, involved in the research.

Study design

In this research, we retrospectively analyzed the effects of the REAC NPO treatment over time. In the practice of the clinical centers affiliated with the Society of Neuro-Psycho-Physical Optimization with REAC technology (SONC), the stability of the disappearance of the FD after the REAC NPO administration is checked every time the patient comes back for control without time programming.

The coordinator of the research launched an initial invitation to the members of SONC, to participate in this research project. Following this invitation, a webinar was organized to illustrate the objectives and how to collect the data.

Clinicians who agreed to participate were asked to provide an anonymized grid, where the following data were reported: gender and age of the patient at the date of the FD assessment, date of the NPO administration, and verification of its effectiveness. Each operator analyzed their database of the patients treated with NPO, and each patient was investigated to verify whether over time the functional dysmetria had recurred in the checks following the administration of the NPO treatment. For the data collection, each patient was entered only once, considering only the control date furthest from the administration of the NPO treatment. This method allows highlighting for each patient the longest stability over time of the effects of NPO on functional dysmetria. The datasheet was collected and sent to the coordinator, then aggregated and subsequently processed.

Population

The data presented in this research were retrospectively collected from a population comprising patients who showed up at the SONC clinical centers involved in the project, over 18 years starting from September 2003.

The data collection concerned a population of 29,794 subjects, 11,981 males and 17,813 females. The average age was 45.07 for males and 50.33 for females. The overall average age was 47.70 (Table 1).

**Table 1:** Population demographics

POPULATION = 29,794	
Males	Females
11,981 (40.21%)	17,813 (59.79%)
AVERAGE AGE OF THE POPULATION = 47.70	
Males	Females
45.07	50.33

In the clinical practice of the SONC centers, all patients undergo the assessment for FD. All the patients showed FD and received the REAC NPO treatment, regardless of the symptomatology, and pathology initially referred.

Functional dysmetria assessment

Functional dysmetria is a phenomenon not perceptible to the subject, but evident to the examiner during the motor task of the assessment maneuver [20,21]. The assessment of FD is an evaluation of neurological semiology aimed at observing the presence of dysfunctional adaptive motor behavior and not a morphological modification.

To assess the presence of FD, the subjects were asked to lie down on the medical examination table and move from the supine to the sitting position [20,21]. During the execution of this motor task, the examinator places his hands lightly on the femoral quadriceps of the subject, and in such a way as to perceive muscle contraction and movement, without opposing and taking care that the nails of his left and right thumbs are perfectly aligned.

In the transition of the subjects from the supine to the sitting position, Rinaldi and Fontani observed a progressive misalignment of the two thumbs. This motor task allows the operator to perceive and highlight the asymmetric activation (dysmetria) of symmetrical muscle groups, such as the quadriceps muscles.

REAC neuro postural optimization treatment

The REAC NPO neuromodulation is a preprogrammed single session treatment. It is administered by applying the tip of the metallic REAC asymmetric conveyer probe (ACP) to a specific area of the ear located at the superior edge of the lower third of the scapha of the ear pavilion, according to the NPO protocol [20,21,23].

Study replicability

The REAC device’s parameter for administering the NPO treatment is set by the manufacturer of the device and cannot be modified by the operators. The methods of administration have been extensively described in previous papers [15,20,21,23].

The models of the equipment used were the CRM, BENE 101, and BENE 110 models, which are equivalent to each other for the administration of REAC NPO. All the devices were produced by ASMED S.r.l, Florence, Italy.

## Results

The data collected on the outcomes of the verification of the stable recovery of the FD are distributed over 18 years and show a higher concentration of cases in the first 36 months after the administration of the NPO treatment, while the cases tend to rarefy as we move away from the date of administration (Table 2).

**Table 2:** Distribution of patients by time slots and percentage of patients with stable disappearance of FD

Months											
Time slot	1	2	3	4	5	6	7	8	9	10	11
Number of patients	2410	2508	976	832	261	1455	272	222	256	153	108
% Stable effects	100%	100%	100%	100%	100%	100%	100%	100%	100%	100%	100%
Years											
Time slot	1	2	3	4	5	6	7	8	9	10	11
Number of patients	3809	3662	2680	1972	1829	1329	732	790	654	637	438
% Stable effects	100%	100%	100%	100%	100%	100%	100%	100%	100%	100%	100%
Time slot	12	13	14	15	16	17	18
Number of patients	424	382	366	277	250	217	189
% Stable effects	100%	100%	100%	100%	100%	100%	100%

This phenomenon can be explained by the physiological tendency to abandonment by patients, who gradually thin out follow-up visits until they don't show up anymore. This explains why out of a total population of 29,794 subjects, the data collected 18 years after the administration of the NPO relate only to 189 subjects (as seen above in Table 2). However, the percentage of patients who presented the stable disappearance of FD is equal to 100% in all the time slots of the data collection (Table 2).

## Discussion

Any environmental interaction, even unconscious, can only take place through the nervous system, regardless of its evolutionary level [36,37]. For this reason, the changes produced by exposome [17], environmental stress, or allostatic overload are present in all living beings [19,36] and can be observed even as morphological asymmetry phenomena [38,39].

This phenomenon is known as FA [1], and in scientific literature, it is described as a morphological asymmetry resulting from errors in the development of normally symmetrical bilateral traits under stressful conditions [2-4].

Based on the phenomenon of FA, which is already considered an epigenetic measure of environmental stress, Rinaldi and Fontani developed the concept of FD, a phenomenon arising upstream of the FA. The FD is an adaptive motor behavior [24] and is detectable with a specific semiological assessment as an asymmetrical activation of symmetrical muscle groups during a motor task. This dysfunctional motor behavior does not originate from lesions or pathologies of the cerebellum, but from a dysfunctional response to environmental stressors by the cerebellum and neural circuits.

From these premises, the authors studied how to act on the nervous system to allow the recovery of the correct adaptive mechanisms and they conceived the radio electric asymmetric conveyer (REAC) technology for neurobiological stimulation treatments, aimed at counteracting the dysfunctional effects induced by the adaptive phenomenon [21,29-35].

Among the REAC neurobiological stimulation protocols, the treatment called neuro postural optimization (NPO) was devised.

In previous studies conducted with fMRI, NPO treatment was able to re-modulate the activation of the brain areas involved in a motor task, increasing the efficiency and competence of the activation pattern, as highlighted by the blood-oxygen-level-dependent (BOLD) signal level modification [20,21,23]. A higher BOLD signal was observed in the brain areas where the greater neural activity requires an increase in oxygen consumption, which in turn is detectable through an increase of deoxyhemoglobin in brain blood flow.

After the NPO treatment, the greater functional efficiency of the brain dynamics is detectable during fMRI through a reduction of BOLD signal in areas not competent for the motor task performed and a reduction in the extension and degree of their activation since the activation is concentrated in specifically competent areas [20,21].

In other previous studies, the NPO treatment has been shown to induce a long-lasting disappearance of FD through the induction of greater functional efficiency in the execution of motor tasks [20,21], even in subjects suffering from neurodegenerative diseases such as Alzheimer's [40-42].

In the present research, a single REAC NPO treatment session showed to be effective in 100% of the treated subjects and the disappearance of FD lasted up to 18 years.

Retrospective studies have important limitations in the quality and quantity of data available for analysis since data was rarely collected per the needs of the study. But in this study the assessment of FD was performed as it would have been done in a prospective study as the characteristics of the subjects included, the data to be collected, and the outcomes to be measured were defined before the study was carried out.

## Conclusions

According to the conceptuality formulated by Rinaldi and Fontani, the verified correlation between the REAC NPO treatment and the stable duration of the disappearance of the FD can be explained as optimization of some functional components of the adaptation processes. The authors correlated the concept of FD as a phenomenon of dysfunctional adaptive origin to the role of the cerebellar functions and other neurodynamics, involved not only in motor control but also in emotional, affective, cognitive, relational control, and stress response.

Based on the hypothesis of the mechanism of action of REAC technology, the NPO treatment is supposed to induce a stable improvement of the neurodynamics and greater efficiency and competence of the brain areas activated in the motor task. This effect would find its clinical demonstration in the long-lasting recovery of FD, likely resulting from a better neurobiological response capacity of the subject to the exposome, and therefore from better control of the symmetry of the adaptive motor behavior.
